# Decreased Interleukin-10 Responses in Children with Severe *Mycoplasma pneumoniae* Pneumonia

**DOI:** 10.1371/journal.pone.0146397

**Published:** 2016-01-11

**Authors:** Shenggang Ding, Xiaowu Wang, Wei Chen, Yuan Fang, Boyu Liu, Yan Liu, Guanghe Fei, Linding Wang

**Affiliations:** 1 Department of Pediatrics, the First Affiliated Hospital of Anhui Medical University, Hefei, China; 2 Department of Microbiology, Anhui Medical University, Hefei, Anhui, China; 3 Department of Respiratory Medicine, First Affiliated Hospital of Anhui Medical University, Hefei, Anhui, China; 4 Department of Clinical Laboratory, Fuyang Second People’s Hospital, Fuyang, Anhui, China; University of Texas Medical Branch, UNITED STATES

## Abstract

Several cytokines may play roles in the immunological pathogenesis of mycoplasmal pneumonia caused by *Mycoplasma pneumoniae*. In this study, we investigated serum cytokine profiles in children with mycoplasmal pneumonia. The serum levels of interleukin (IL)-8, IL-10, and IL-18 were examined using ELISA kits in 34 patients with *M*. *pneumoniae* infection (Group 1, 11 with severe mycoplasmal pneumonia; Group 2, 13 with mild mycoplasmal pneumonia; Group 3, 10 with asthma) and 32 age-matched, non-infected controls. The serum levels of IL-8, IL-10, and IL-18 increased significantly in patients with mycoplasmal pneumonia compared with those in controls (P<0.01). The serum levels of IL-10 decreased significantly in Group 1 compared with those in Group 2 (P<0.01). The serum levels of IL-18 increased significantly in Group 1 compared with those in Group 2 (P<0.01). The serum levels of IL-10 and IL-18 decreased significantly in 10 *M*. *pneumoniae*-infected patients with asthma compared with those in 24 *M*. *pneumoniae*-infected patients without asthma (P<0.01). We examined the level of interleukins (IL-8, IL-10 and IL-18) after the patients started therapy. The data showed that IL-18 were lower after therapy (P<0.01). Collectively, our data suggested that these cytokines may be involved in the pathogenesis of mycoplasmal pneumonia.

## Introduction

*Mycoplasma pneumoniae* is recognized as one of the most important pathogens in community-acquired pneumonia (CAP) in young adults and children [1]. *Mycoplasma pneumoniae* gives rise to mycoplasmal pneumonia, also called atypical pneumonia, which has been shown by many physicians to have different clinical features. It accounts for 7–20% of CAP [2]. Many studies showed that atypical pneumonia might have a higher incidence in patients with mild symptoms, and atypical pneumonia is not only considered to cause respiratory disease, but also severe diseases including neurological, dermatological, hematological, renal, gastrointestinal, and musculoskeletal diseases [3]. Further reports showed that the clinical parameters of atypical pneumonia were different in patients without *M*. *pneumonia* pneumonia, and symptoms include fevers, crackles, thrombocytosis, consolidation, sore throat, headache, skin rash, ear infection. Although the pathogenesis of mycoplasmal pneumonia is not clear at present, these manifestations may result from the immune response to infection and/or the ability of *M*. *pneumoniae* to directly invade the ciliated airway epithelial cells in the respiratory tract [2–4].

Researchers have found that the cell-mediated immune response of the host plays an important role in the mechanism of mycoplasmal pathogenicity, specifically Th1-type cytokines [5]. IL-8, IL-18 and IL-10 level in serum were higher in people with mycoplasmal pneumonia [6–13]. Measurement of their levels can help understand conditions of the patients. Recent investigations have shown that *M*. *pneumoniae* lysates may increase interleukin-8 (IL-8) levels in human airway epithelial cells, may contribute to neutrophilic asthma [5, 6, 8]. Several studies showed that *M*. *pneumoniae* infection in children induced IL-18, which may be a central factor in the immunopathologic response [10, 11, 13]. IL-18, a Th1-type cytokine, was originally designated interferon gamma (IFN-γ)-inducing factor. This cytokine was first reported to be produced by Kupffer cells and activated macrophages, and it was shown to be a critical factor in inducing liver injury in mice [9]. IL-18 serums level in adult with severe mycoplasmal pneumonia was significantly higher than those in patients with mild cases, suggesting that pulmonary lesions may be modulated in *M*. *pneumoniae* infected humans [13]. Chung et al. studied 75 children with mycoplasmal pneumonia and divided them into asthmatic and non-asthmatic groups. They found that children with asthma exhibited a deficient IL-18 response and had more severe pneumonia [6]. Oishi et al. measured serum IL-18 level in 23 acute or convalescent patients and demonstrated that there was a significant correlation between the level of IL-18 and lactate dehydrogenate (LDH) in serum [11]. They thought that the LDH level can be used to estimate the IL-18 level. A gnotobiotic mouse model of *M*. *pneumoniae* pneumonia was established, which showed that cytokines, including IL-4, IL-10 and IFN-γ, were significantly increased in mice that were repeatedly infected with *M*. *pneumonia* [7, 12]. However, few researchers have examined IL-10 level in serum in severe *M*. *pneumoniae* infections in children patients. The aim of this study was to investigate IL-10 level in the serum of children infected with *M*. *pneumoniae* pneumonia. At the same time, the correlation between the cytokines (IL-8, IL-10 and IL-18) and the other clinical factors was analyzed. We found that the serum levels of IL-10 decreased significantly in patients with severe mycoplasmal pneumonia compared with those with mild mycoplasmal pneumonia (P<0.01). The serum levels of IL-10 and IL-18 decreased significantly in 10 *M*. *pneumoniae*-infected patients with asthma compared with those in 24 *M*. *pneumoniae*-infected patients without asthma (P<0.01). Our data showed that both IL-8 and IL-18 levels were positively correlated with CRP levels. Our findings suggest that CRP level can be used to estimate the levels of IL-8 and IL-18. The levels of IL-10 and IL-18 could help diagnose the severity of pediatric *M*. *pneumoniae* pneumonia.

## Materials and Methods

### Study Subjects

We recruited 34 children with mycoplasmal pneumonia, including 20 girls and 14 boys, aged 1–12 years, (median age 5 years), who were admitted to the First Affiliated Hospital of Anhui Medical University between August 2013 and April 2014. We enrolled 32 age-matched, non-*M*. *pneumniae* infected children as controls. The 7 serum samples after therapy were collected. The diagnosis of pneumonia in all patients was judged on both clinical and radiological findings. *M*. *pneumniae* infection was confirmed by a serologic test (IgM titer ≥ 1∶160) using the Serodia Myco II particle agglutination test (Fujirebio, Tokyo, Japan,). Acute blood sample was taken from each patient within 7 days from the onset of the symptoms. Serum samples were stored at -70°C until they were assayed for IL-8, IL-10, and IL-18. The disease severity was defined as sores from 0 to 5 based on the following clinical findings according to the previous studies with modification [14, 15]: rapid breathing or lower chest wall in drawing, fever (>38.5°C), more than 2 affected pulmonary lobes on chest X-rays, more than 7 days of hospital stay.

The patients with severity score≥3 was defined as severe pneumonia group and ≤2 as mild pneumonia group. Asthma was defined with the physician’s diagnosis of bronchial hyperresponsiveness on at least 12% of reversibility of EFV1 after β2 agonist inhalation or methacholine challenge [6]. The patients were divided into three groups: those who had severe cases of *M*. *pneumoniae* pneumonia without asthma (Group 1, n = 11), those who had mild cases of *M*. *pneumoniae* pneumonia without asthma (Group 2, n = 13), and those who were diagnosed with *M*. *pneumoniae* pneumonia with asthma (Group 3, n = 10). Informed written consents were obtained from all parents and permission to conduct the study was approved by the Ethics Committee of Anhui Medical University.

### Cytokine Assays

Serum cytokine levels of IL-8, IL-10, and IL-18 were determined using Human IL-8, IL-10 and IL-18 ELISA Kit (Shanghai Yuanye Bio-Technology, Shanghai, China) following the manufacturer’s instruction. The minimal significant level detected was 12.5 pg/ml for IL-8 and IL-18, and 1.0 pg/ml for IL-10, as set by the manufacturer.

### Clinical Laboratory Data

White blood cell (WBC) counts, neutrophil (NEU) counts, C-reactive protein (CRP) levels, lactate dehydrogenate (LDH) levels, lymphocyte counts, and red blood cell (RBC) counts were performed by routine methods. Demographic data were obtained from the children’s parents.

### Statistical Analysis

Data on serum IL-8, IL-10, and IL-18 levels were expressed as means ± standard deviation (SD). Sixty-six children were classified into 34 patients with mycoplasmal pneumonia and 32 patients without mycoplasmal pneumonia. The 34 patients with mycoplasmal pneumonia were classified into three groups. IL-8, IL-10, and IL-18 levels were compared among patients with mycoplasmal pneumonia and control subjects using a Student’s *t*-test, as the data were normally distributed. RBC counts, WBC counts were compared using chi-squared tests, and their counts were also shown with means ± SD. Neutrophil counts, CRP levels, LDH levels, and lymphocyte counts, which were not normally distributed, were compared using Mann-Whitney U tests, and their levels were shown with interquartile range (IQR). The relationship between IL-8, IL-10, IL-18, and laboratory data were analyzed using Pearson’s product moment correlation coefficient. A two-sided P value <0.05 was considered significant. The results of the analysis were obtained using SPSS for windows.

## Results

**Fig 1:**
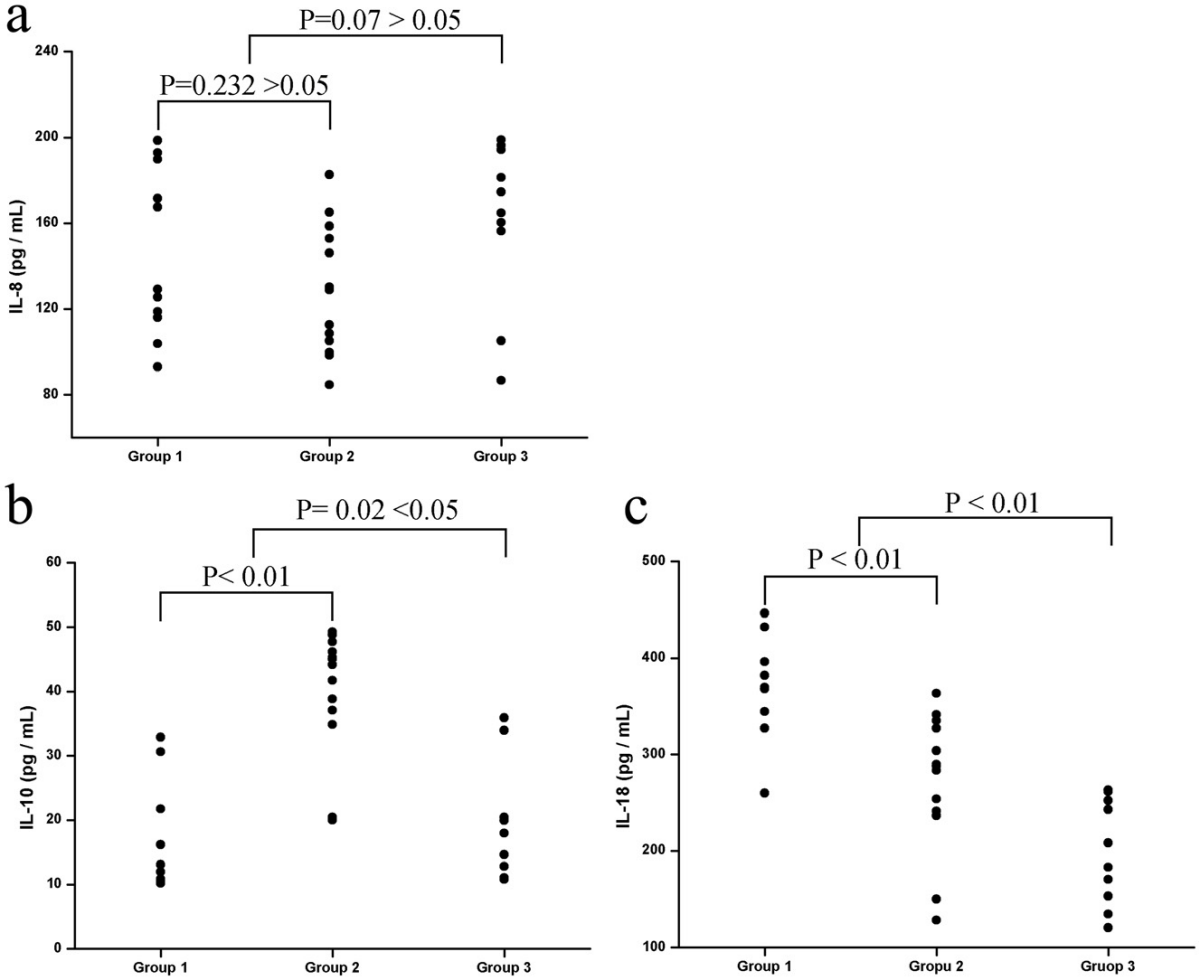
IL-8 (a), IL-10 (b) and IL-18 (c) levels in non-asthmatic patients with severe *M*. *pneumoniae* pneumonia (group 1) and mild *M*. *pneumoniae* pneumonia (group 2), and in asthmatic patients with *M*. *pneumoniae* pneumonia (group 3). Serum IL-10 and IL-18 levels were significantly higher in patients infected by *M*. *pneumoniae* without asthma than those in group 3. However, IL-8 levels were not significantly different. Serum IL-10 levels were significantly lower in group 1 than in group 2, while IL-18 levels were significantly higher in group 1; IL-8 levels were not significantly different.

The characteristics, demographic data, serum IL-8, IL-10, and IL-18 levels of patients with mycoplasmal pneumonia and control subjects are shown in Table 1. There were no significant differences in WBC counts, LDH levels, lymphocyte counts, and RBC counts between patients with mycoplasmal pneumonia and control subjects. IL-8, IL-10, and IL-18 levels of patients before therapy and after therapy are shown in Table 2. Patients with mycoplasmal pneumonia had significantly higher CRP, IL-8, IL-10, and IL-18 levels than control subjects. However, control subjects had significantly higher NEU counts than patients with mycoplasmal pneumonia. The IL-8, IL-10, and IL-18 levels of the 34 patients with mycoplasmal pneumonia are shown in Fig 1. The levels of IL-8 in Group 1 (mean ± SD: 146.0 ± 36.9 pg/ml) and Group 2 (mean ± SD: 128.7 ± 29.0 pg/ml) were not significantly different (P = 0.232). IL-8 levels did not significantly differ between Group 3 (mean ± SD: 161.9 ± 36.1 pg/ml; P = 0.07) and the 24 *M*. *pneumoniae*-infected patients without asthma (mean ± SD: 136.6 ± 33.9 pg/ml) (Fig 1a). There were significantly lower levels of IL-10 in Group 1 (mean ± SD: 16.8 ± 7.8 pg/ml; P<0.0001) compared with Group 2 (mean ± SD: 39.9 ± 9.4 pg/ml). The study also found lower levels of IL-10 in Group 3 (mean ± SD: 19.0 ± 8.6 pg/ml) compared with the 24 *M*. *pneumoniae*-infected patients without asthma (mean ± SD: 29.4 ± 14.4 pg/ml), which was significantly different (P = 0.02) (Fig 1b). The IL-18 level of Group 1 (mean ± SD: 374.4 ± 48.9 pg/ml) was significantly different from that of Group 2 (mean ± SD: 272.5 ± 67.9 pg/ml; P = 0.001). The IL-18 level of Group 3 (mean ± SD: 199.1 ± 51.2 pg/ml; P<0.01) was significant lower than that of the 24 *M*. *pneumoniae*-infected patients without asthma (mean ± SD: 319.2 ± 78.5 pg/ml) (Fig 1c).

**Table 1:** Clinical factures, laboratory findings, and serum levels of IL-8, IL-10 and IL-18 of patients with *M*. *pneumoniae* pneumonia and control subjects.

	Patients with M. *pneumoniae* pneumonia (n = 34)	Control subjects (n = 32)	P Values
WBC (10^9^/L)	8.3 ± 3.0	6.3 ± 1.6	0.104
NEU (%)	46.4	14.5	0.032
Lymphocyte counts (%)	38.9	13.3	0.164
RBC (10^12^/ L)	4.4 ± 0.5	4.6 ± 0.3	0.554
CRP (g/ml)	27.9	3.9	<0.001
LDH (pg/ml)	32.7	28.7	0.410
IL-8 (pg/ml)	144.1 ± 36.5	116.8 ± 19.5	0.044
IL-10 (pg/ml)	26.3 ± 13.8	8.8 ± 5.9	<0.001
IL-18 (pg/ml)	283.9 ± 90.1	162.4 ± 20.1	<0.001

Data on serum IL-8, IL-10, IL-18, WBC, and RBC are presented as means ± SD; Date on Neutrophil counts, CRP levels, LDH levels, and lymphocyte counts are shown with IQR.

**Table 2:** Serum levels of IL-8, IL-10 and IL-18 of patients before therapy and after therapy.

	Before therapy	After therapy	P Values
IL-8 (pg/ml)	139.1 ± 31.5	110.8 ± 19.5	0.576
IL-10 (pg/ml)	30.3 ±13.7	10.8 ± 4.3	0.514
IL-18 (pg/ml)	249.9 ± 40.9	97.5 ± 19.1	0.020
CRP (g/ml)	48.4±21.3	4.5±3.2	0.001

Data are presented as means ± SD.

**Fig 2:**
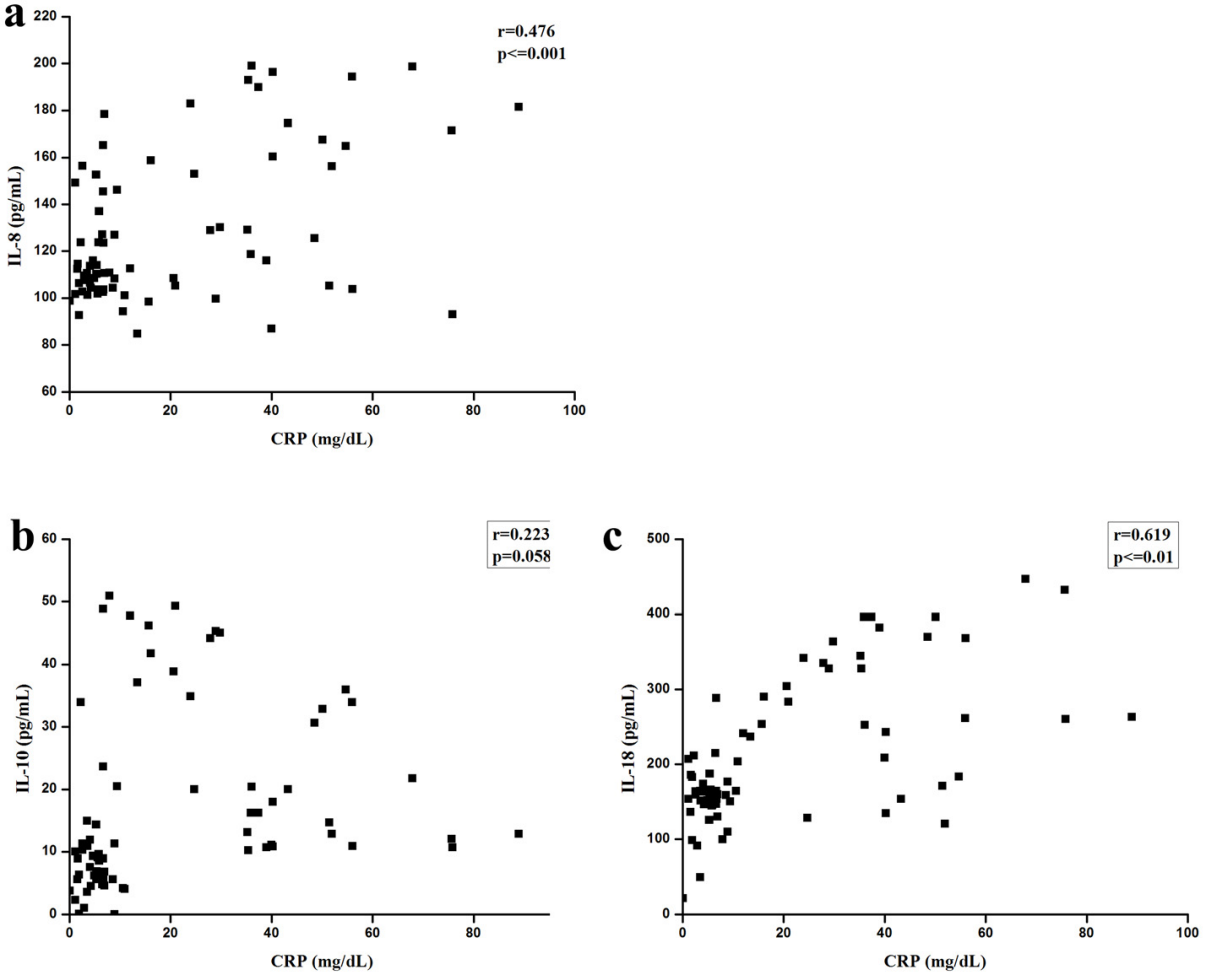
Serum levels of IL-8 (a), IL-10 (b), and IL-18 (c) relationship with CRP. Significant relationships were found between IL-8 levels and CRP levels, as well as between IL-18 levels and CRP levels, although no significant relationship was found between IL-10 and CRP levels.

The correlations between IL-8, IL-10, and IL-18 levels, and clinical parameters CRP levels are shown in Fig 2. Meanwhile, the correlation between the levels of IL-8 and CRP (r = 0.476, P< = 0.001), as well as the correlation between the levels of IL-18 and CRP (r = 0.619, P< = 0.001), were also statistically significant, although the degree of the relationships was not very strong (Fig 2a and 2c). The correlation between IL-10 and CRP was not significant (Fig 2b). However, significant correlations were not found between IL-8, IL-10, and IL-18 levels, and WBC counts, lymphocyte counts, RBC counts, NEU counts, and LDH levels (S1–S4 Figs).

## Discussion

*M*. *pneumoniae*, which lacks a peptidoglycan cell wall, is one of the extracellular pathogens that cause community-acquired respiratory infections. PCR methods can be used to detect *M*. *pneumoniae* in sputum, throat swabs, or bronchoalveolar lavage fluid, as *M*. *pneumoniae* attaches to ciliated airway epithelial cells of the respiratory tract. PCR methods have a high sensitivity to detect microbial nucleic acids. However, positive PCR results cannot confirm reliably that the organism is alive or infectious [16, 17]. Some studies showed that levels of secreted cytokines, such as IL-2 and IL-5, were significantly different in patients infected by *M*. *pneumoniae* compared with other patients [18, 19]. Pang et al. reported that the TNF-α, IL-6 and IL-10 levels in children with M. pneumoniae pneumonia were higher at acute stage than those in the control group, suggesting the cytokines maybe involved in the pathogenesis of M. pneumoniae pneumonia [20]. Delayed-type hypersensitivity skin reaction responses to *M*. *pneumoniae* antigens appear to be correlated with the severity of pneumonia. Serum IL-18 and sIL-2R levels in patients with severe *M*. *pneumoniae* infection group were higher than those in patients with mild infections, and there was a significant relationship between them [13]. Oishi et al. pointed out that most hospitals and clinics cannot usually measure IL-18 secretion levels quickly, so it is important to search for correlations between IL-18 levels and other clinical features [11]. But if the condition permits, cytokines detection is necessary. Transgenic IL-10-deficient mice can spontaneously acquire a chronic inflammatory bowel disease. The finding indicated that endogenous IL-10 is important to inhibit the inflammatory response, while IL-10 has a dual role in the inflammatory bowel disease [21]. IL-10 is expressed in the lungs of *M*. *pneumoniae*-infected BALB/c mice, and it is believed that IL-10 serves to turn off the specific T-cell response [10, 12]. In our study, serum IL-10 levels were higher in patients with mycoplasmal pneumonia, which is in accordance with the findings of previous studies [16, 22]. Mizukane et al. reported an extremely rare case of a 76-year-old Japanese who developed hemophagocytic syndrome due to fulminant *M*. *pneumoniae* pneumonia, and they found that elevated sIL-2R, IL-6, and IL-10 levels may be caused by severe mycoplasmal pneumonia [9]. We examined three groups of patients with mycoplasmal pneumonia, including severe and mild patients without asthma and patients with mycoplasmal pneumonia with asthma. In severe cases, IL-10 levels were lower than in mild cases. This result can be explained by the fact that the ability of IL-10 to inhibit inflammation may be decreased by some factors. Recently, researchers have suggested that the onset of asthma may be preceded by *M*. *pneumoniae* infections, or that asthma symptoms may be aggravated by infecting *M*. *pneumonia* [23]. In cases with asthma, IL-10 levels were lower, which suggests that some asthmatic children have deficient IL-10 responses when infected by *M*. *pneumoniae*. Seo YH et al reported the levels of interleukin-18 in nasopharyngeal aspirate were significantly higher in patients infected with *Mycoplasma pneumoniae* who did not respond to macrolide treatment[24]. We also detected other chemokines, including IL-8, IL-18, and other clinical factors. Serum IL-8 levels in patients with *M*. *pneumoniae* were significantly higher than those in control subjects, and IL-8 was induced in airway epithelial cell line A549 by a *M*. *pneumoniae* lysate (MPL), which may have increased neutrophil chemotaxis and ERK activity [8]. Kyung et al. reported that MPL stimulated the production of IL-8 in human airway epithelial cells [23]. Narita et al. detected IL-8 levels in pleural fluid samples and serum samples from patients with *M*. *pneumoniae* infections, and the data showed that IL-8 levels were significantly elevated in pleural fluid and serum [25]. Our results are in agreement with the view that serum IL-8 may be involved in the pathogenesis of the pulmonary disease features of *M*. *pneumoniae* pneumonia. Our data showed that IL-8 levels were not significantly different in patients with or without asthma, which indicates that IL-8 may not play a pivotal role in asthma. IL-18 levels in patients with mycoplasmal pneumonia were significantly higher than those in control subjects, and they were lower in patients with asthma compared with patients without asthma. Serum IL-18 levels in children in our study were similar to those of previous reports in adults and children [6, 13]. These results suggest that these chemokines play an important role in the pathogenesis of respiratory infections.

Seo YH et al also found that the levels of CRP in nasopharyngeal aspirate were significantly higher in patients infected with *Mycoplasma pneumoniae* who did not respond to macrolide treatment. They suggested that serum CRP could be a useful marker for predicting the efficacy of macrolides and helping clinicians make better clinical decisions in children with macrolide-resistant *Mycoplasma pneumoniae* [24]. We also analyzed the clinical characteristics of the study subjects, including CRP Table 1 shows that CRP levels were higher and NEU counts were lower in patients with *M*. *pneumonia* infection. We analyzed the correlation between IL-8, IL-10, and IL-18, and the clinical factors. The cause of this result may be due to different cell-mediated immune responses of the host. Our data showed that both IL-8 and IL-18 levels were positively correlated with CRP levels. An association of IL-10 levels and clinical factors was not found. Table 2 shows that IL-18 levels were lower after therapy (P = 0.020), CRP levels were also significant lower after therapy (P = 0.001). But after therapy, the correlation between the levels of IL-8, IL-18 and CRP was not significant. (S5 Fig) In summary, our data indicate that IL-8, IL-10, and IL-18 might modulate pulmonary lesions in mycoplamal pneumonia.

## Conclusions

Our findings suggest that CRP level can be used to estimate the levels of IL-8 and IL-18. If necessary, the levels of IL-10 and IL-18 should be measured, as they can help diagnose the severity of pediatric *M*. *pneumoniae* pneumonia.

## Supporting Information

S1 Fig Serum levels of IL-8 (a), IL-10 (b), and IL-18 (c) relationship with RBC counts. (TIF)

S2 Fig Serum levels of IL-8 (a), IL-10 (b), and IL-18 (c) relationship with NEU. Significant relationships were not found between them and NEU, respectively.
(TIF)

S3 Fig Serum levels of IL-8 (a), IL-10 (b), and IL-18 (c) relationship with Lymphocyte counts. (TIF)

S4 Fig Serum levels of IL-8 (a), IL-10 (b), and IL-18 (c) relationship with WBC counts. (TIF)

S5 Fig Serum levels of IL-8 (a), IL-18 (b) relationship with CRP after therapy. (TIF)
