# Supplementary figures and images for: Decreased Interleukin-10 Responses in Children with Severe Mycoplasma pneumoniae Pneumonia
Source: PLoS One. 2016 Jan 11;11(1):e0146397. doi: 10.1371/journal.pone.0146397 (PMC4708986; doi:10.1371/journal.pone.0146397)

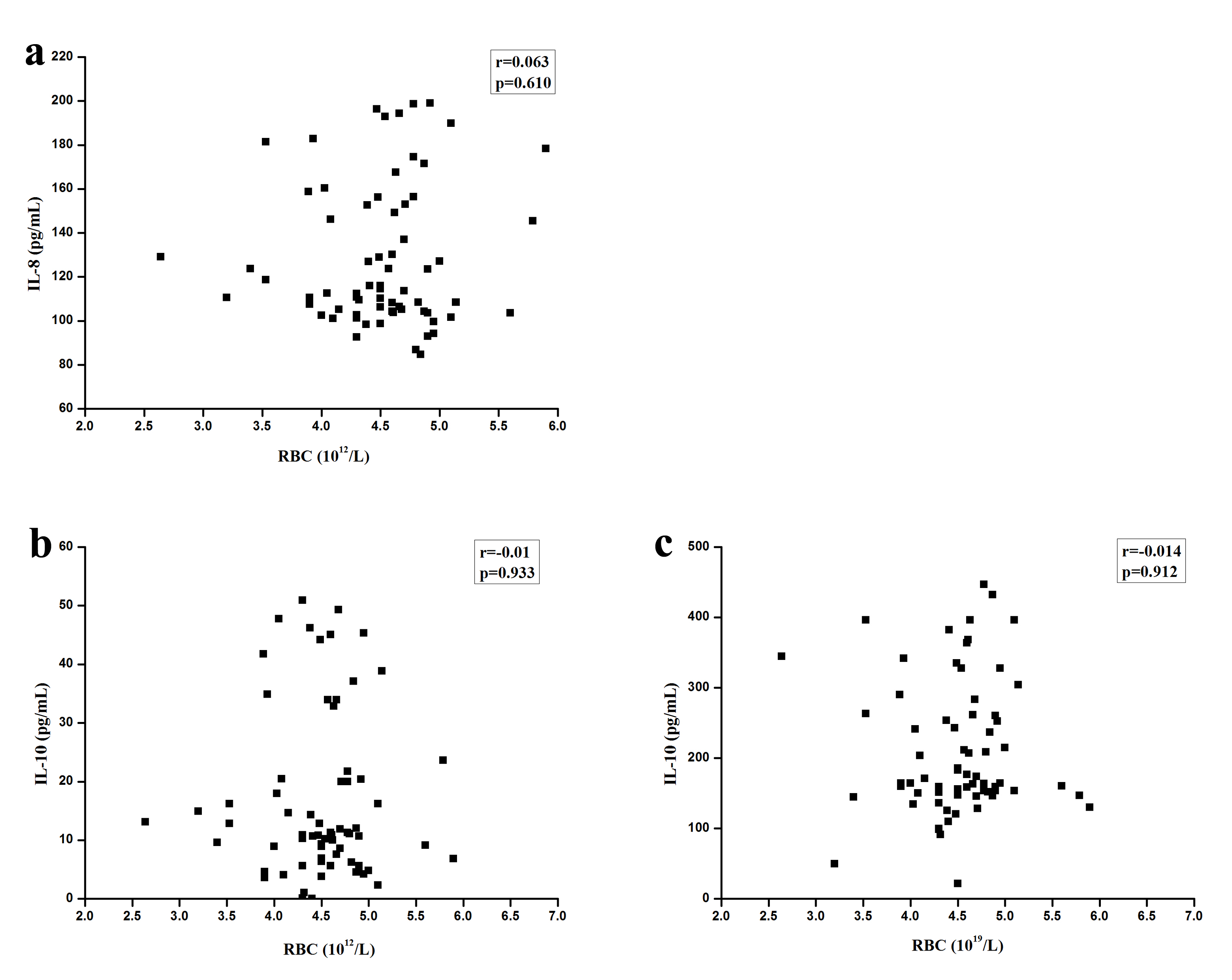

Supplement: S1 Fig — (TIF) [file pone.0146397.s001.tif]

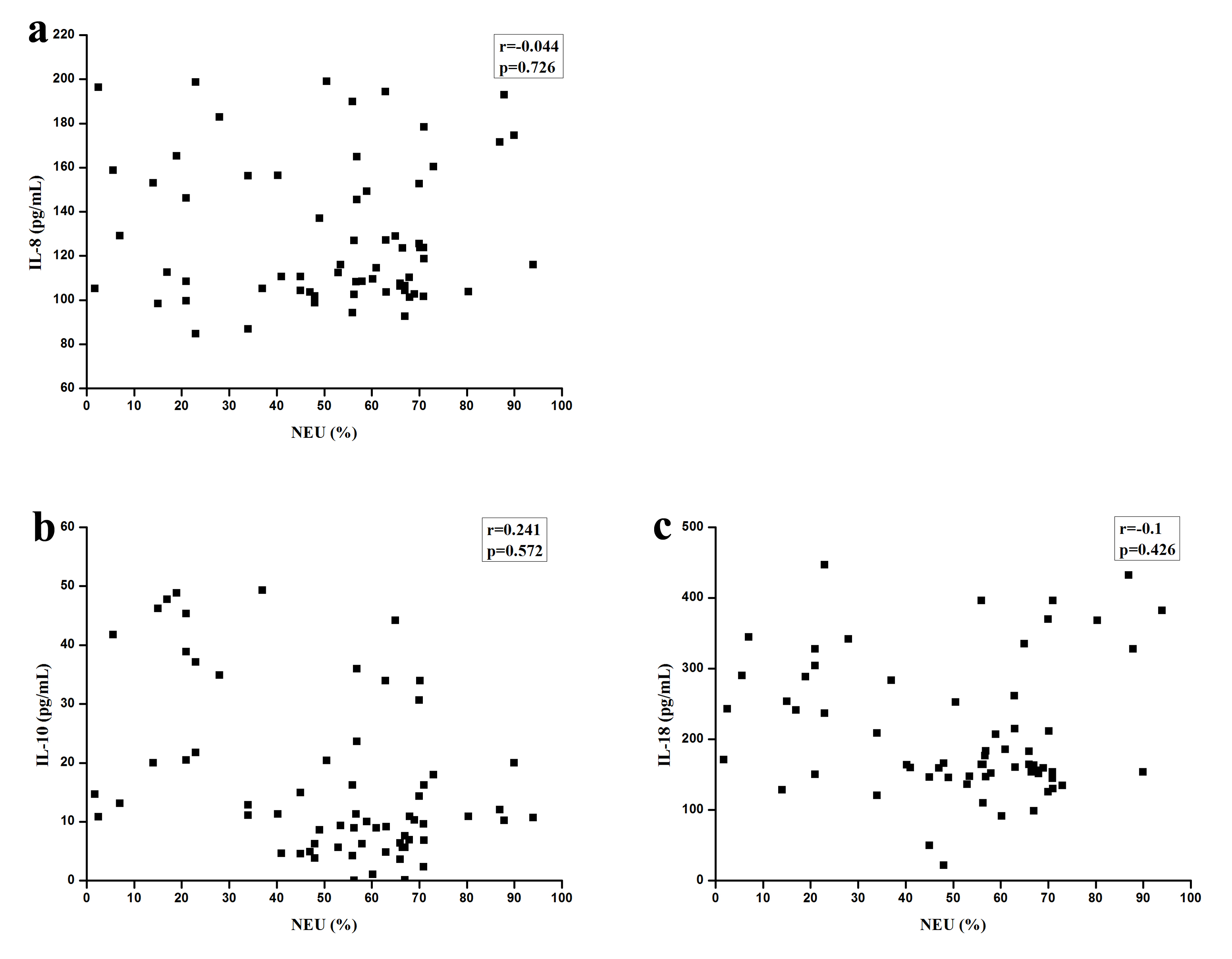

Supplement: S2 Fig — Significant relationships were not found between them and NEU, respectively. (TIF) [file pone.0146397.s002.tif]

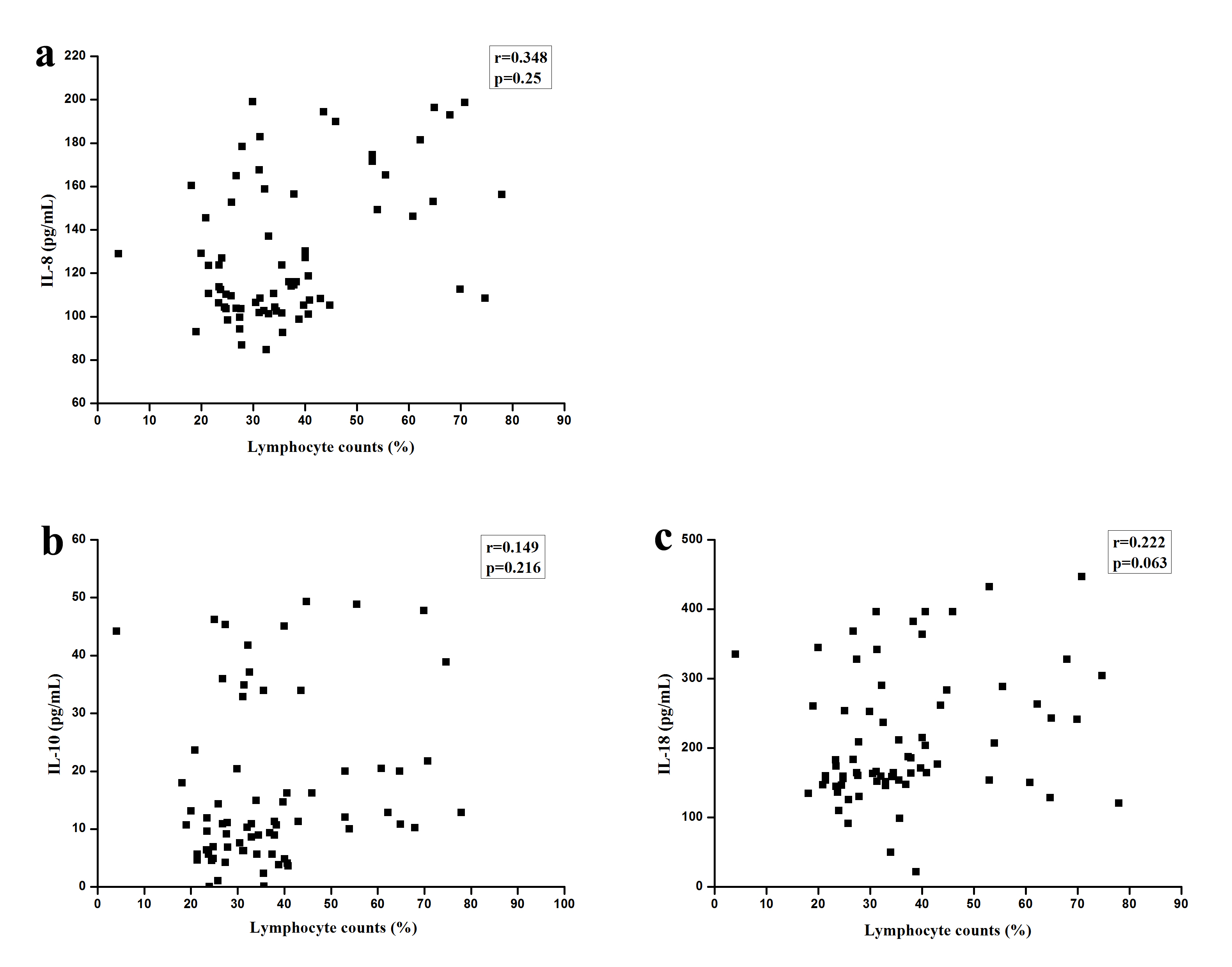

Supplement: S3 Fig — (TIF) [file pone.0146397.s003.tif]

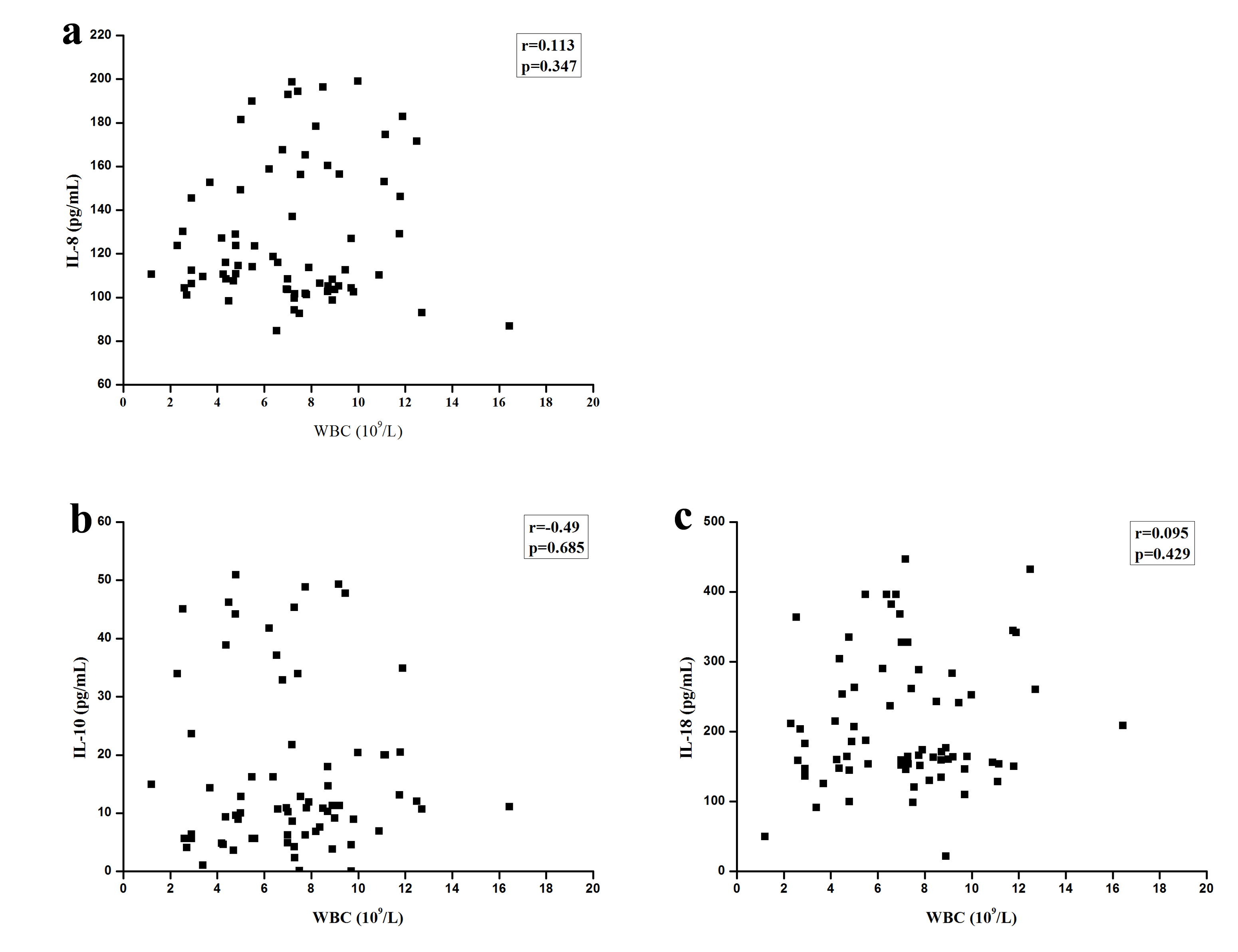

Supplement: S4 Fig — (TIF) [file pone.0146397.s004.tif]

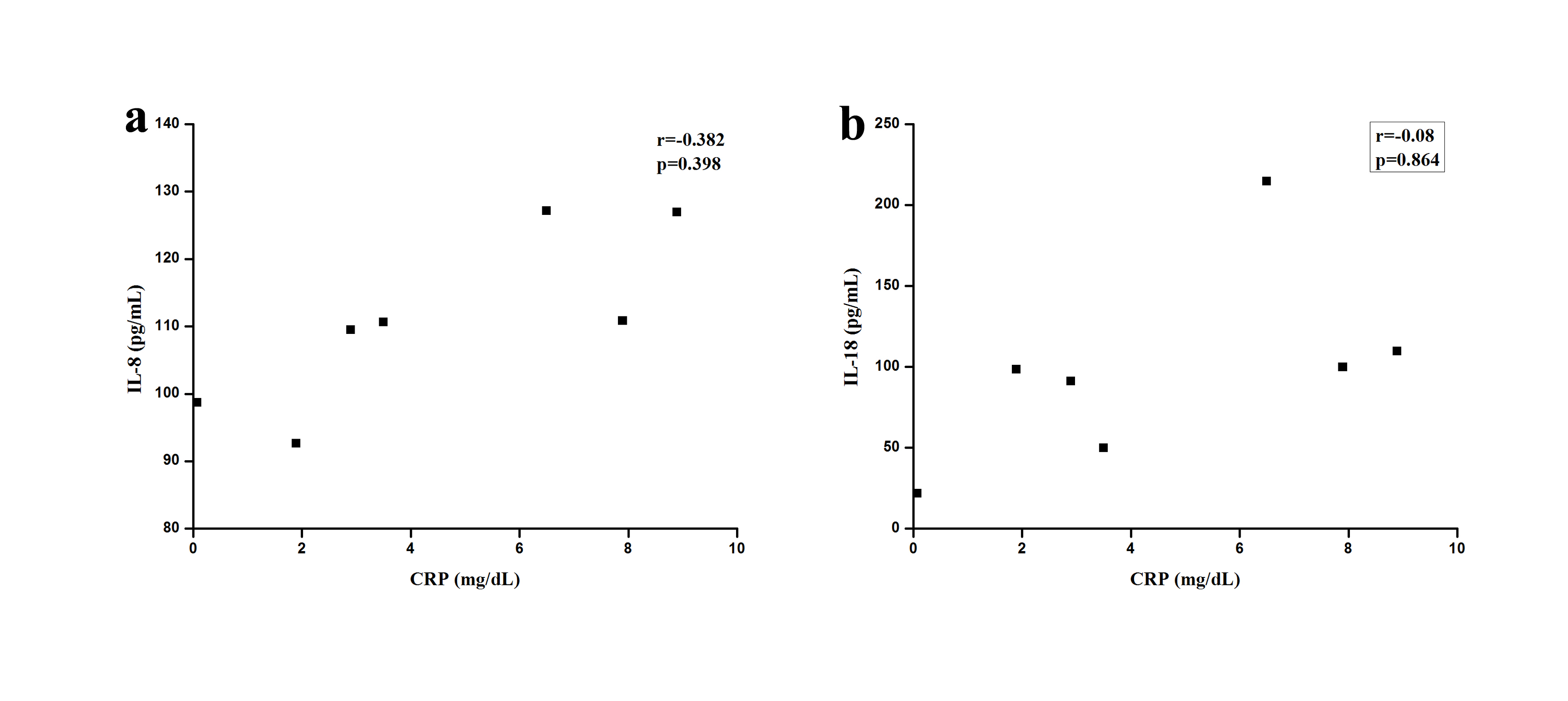

Supplement: S5 Fig — (TIF) [file pone.0146397.s005.tif]
